# Metabolite Fruit Profile Is Altered in Response to Source–Sink Imbalance and Can Be Used as an Early Predictor of Fruit Quality in Nectarine

**DOI:** 10.3389/fpls.2020.604133

**Published:** 2021-01-08

**Authors:** María Paz Covarrubias, Victoria Lillo-Carmona, Lorena Melet, Gianfranco Benedetto, Diego Andrade, Mickael Maucourt, Catherine Deborde, Claudia Fuentealba, Annick Moing, María Luisa Valenzuela, Romina Pedreschi, Andréa Miyasaka Almeida

**Affiliations:** ^1^Departamento de Biología, Facultad de Ciencias, Centro de Biología Molecular Vegetal, Universidad de Chile, Santiago, Chile; ^2^Centro de Biotecnología Vegetal, Facultad de Ciencias de la Vida, Universidad Andrés Bello, Santiago, Chile; ^3^Centro de Genómica y Bioinformática, Facultad de Ciencias, Universidad Mayor, Huechuraba, Chile; ^4^Escuela Ingeniería en Biotecnología, Facultad de Ciencias de la Vida, Universidad Andrés Bello, Santiago, Chile; ^5^Centre INRAE de Nouvelle Aquitaine Bordeaux, MetaboHUB, INRAE 2018, Bordeaux Metabolome, UMR 1332, Biologie du Fruit et Pathologie, Universit de Bordeaux, INRAE, Bordeaux, France; ^6^Escuela de Agronomía, Facultad de Ciencias Agronómicas y de los Alimentos, Pontificia Universidad Católica de Valparaíso, Quillota, Chile; ^7^Inorganic Chemistry and Molecular Material Center, Instituto de Ciencias Químicas Aplicadas, Universidad Autónoma de Chile, Santiago, Chile; ^8^Escuela de Agronom a, Facultad de Ciencias, Universidad Mayor, Huechuraba, Chile

**Keywords:** *Prunus persica*, thinning, sugars, phenylpropanoid, organic acids

## Abstract

Peaches and nectarines [*Prunus persica* (L.) Batsch] are among the most exported fresh fruit from Chile to the Northern Hemisphere. Fruit acceptance by final consumers is defined by quality parameters such as the size, weight, taste, aroma, color, and juiciness of the fruit. In peaches and nectarines, the balance between soluble sugars present in the mesocarp and the predominant organic acids determines the taste. Biomass production and metabolite accumulation by fruits occur during the different developmental stages and depend on photosynthesis and carbon export by source leaves. Carbon supply to fruit can be potentiated through the field practice of thinning (removal of flowers and young fruit), leading to a change in the source–sink balance favoring fruit development. Thinning leads to fruit with increased size, but it is not known how this practice could influence fruit quality in terms of individual metabolite composition. In this work, we analyzed soluble metabolite profiles of nectarine fruit cv “Magique” at different developmental stages and from trees subjected to different thinning treatments. Mesocarp metabolites were analyzed throughout fruit development until harvest during two consecutive harvest seasons. Major polar compounds such as soluble sugars, amino acids, organic acids, and some secondary metabolites were measured by quantitative ^1^H-NMR profiling in the first season and GC-MS profiling in the second season. In addition, harvest and ripening quality parameters such as fruit weight, firmness, and acidity were determined. Our results indicated that thinning (i.e., source–sink imbalance) mainly affects fruit metabolic composition at early developmental stages. Metabolomic data revealed that sugar, organic acid, and phenylpropanoid pathway intermediates at early stages of development can be used to segregate fruits impacted by the change in source–sink balance. In conclusion, we suggest that the metabolite profile at early stages of development could be a metabolic predictor of final fruit quality in nectarines.

## Introduction

Peaches and nectarines [*Prunus persica* (L.) Batsch] are among the most important fruit crops with a world annual production of approximately 25 million tons (Food and Agriculture Organization of the United Nations (FAOSTAT), 2018). They belong to the Rosaceae family, whose species have developed a wide array of fruit types, including drupe, pome, drupetum, achene, and achenetum. *P. persica* displays a drupe-type fruit, where a fleshy juicy mesocarp encloses a lignified endocarp surrounding a seed. The presence of this lignified endocarp imposes a challenge to the proper fruit development and ripening, since the phenylpropanoid allocation must be carefully controlled for the endocarp lignification during development and the biosynthesis of flavor/aroma compounds in the ripe fruit mesocarp (Dardick and Callahan, 2014). The main difference between peaches and nectarines is the absence of trichrome in the surface of nectarines. *P. persica* is a climacteric fruit in which development and ripening are coordinated processes involving physiological, molecular, and biochemical changes (Moing et al., 1998; Lombardo et al., 2011). Fruit growth in this species follows a double sigmoidal curve with four stages clearly defined (S1–S4; Chalmers and van den Ende, 1975; Tonutti et al., 1991). The first stage (S1) corresponds to the first exponential growth due to an increase in cellular division and elongation. The second stage (S2) corresponds to endocarp lignification, which leads to an arrest in growth rate due to the high carbon and energy demand to sustain phenylpropanoid pathway (Dardick et al., 2010). In the third stage (S3), known as the second exponential growth, fruit growth is mainly due to cell enlargement because of water entrance. At the end of S3, the fruit reaches its final size and its background is green. At this moment, the fruit enters the fourth stage (S4) and can be harvested (Ognjanov et al., 1995; Lombardo et al., 2011). The S4 stage corresponds to the ripening stage. At S4 I, fruit is no longer inflated and does not release ethylene. At S4 II, fruit releases little ethylene. At S4 III, ethylene autocatalytic production increases and fruit rapidly softens (Pan et al., 2015). Peaches and nectarines as climacteric fruit show a rise in respiration and ethylene biosynthesis rates at the beginning of ripening (Carrari and Fernie, 2006; Cherian et al., 2014; Karlova et al., 2014). Ethylene induces changes in color, texture, flavor, and aroma, which all together improve the fruit nutritional value and attractiveness promoting its consumption and seed dispersal (Liu et al., 2004; Goff and Klee, 2006).

Fruit development and growth depend on photoassimilates imported from source leaves. In Rosaceae species such as peaches and nectarines, sucrose and sorbitol are the sugars translocated from source leaves to the fruit, which are non-autotrophic sink organs (Lo Bianco et al., 1999). The amount of sugars that will arrive in a fruit depends on the force attracting these “translocated sugars” known as sink strength. The competition with other sink organs (other fruits) also affects the partitioning of carbon in a tree. Once the translocated sugars are unloaded from phloem, they are metabolized into glucose and fructose in the case of sucrose, and into fructose in the case of sorbitol. These hexoses can be used to sustain aerobic respiration or they can be derived to other metabolic pathways for the synthesis of structural carbohydrates, amino acids, and other biomolecules related to growth and development (Génard et al., 2003). Fruit quality is strongly related to metabolite composition and balance (Colaric et al., 2005). Indeed, taste is mainly dependent on the balance of organic acids-to-sugar ratio, conferring acidity and sweetness, respectively (Kader, 2008; Brasil and Siddiqui, 2018). Accumulation of soluble sugars and organic acids related to organoleptic properties occurs at the late stages of fruit development and may occur through direct phloem unloading and through interconversion of metabolites by gluconeogenesis (Génard et al., 2003).

Agronomical practices like thinning consist in the removal of fruits or flowers in order to modify the source–sink balance favoring growth and advancing harvest of the fruits that remain on the tree (Grossman and DeJong, 1995; Link, 2000; Lesičar et al., 2016). Thinning is often performed in commercial orchards to increase final fruit size. The recommendation is to perform thinning in early fruit development stages to take advantage of the availability of photoassimilates (Grossman and DeJong, 1995; Costa and Vizzotto, 2000; Reighard et al., 2018; Sutton et al., 2020). Nevertheless, when thinning is performed too early, productivity may be affected by spring frosts (Byers and Marini, 1994).

Thinning practice is known to improve fruit size and in some cases also total soluble solids (TSS) content (Costa et al., 2018), but it is not clear how this practice could influence fruit quality in terms of individual metabolite composition such as major soluble sugars (Wu et al., 2011). In this work, we analyzed the primary metabolite profile of nectarine fruits cv “Magique” at different developmental stages and from trees subjected to different thinning treatments. Mesocarp polar metabolites were analyzed throughout development until harvest during two consecutive harvest seasons. In addition, harvest and ripening quality parameters such as fruit weight, firmness, acidity, and TSS content were measured. The aim of this work was to evaluate if thinning could alter the metabolic profile of fruits.

## Materials and Methods

### Plant Material and Thinning Treatments

The experiments were performed using 4 year-old early harvest nectarine trees [*P. persica* (L.) Batsch var. “Magique”] from the commercial orchard “Viveros El Tambo” located in El Tambo, O’Higgins Region of Chile (34°28′30.4″S 70°59′07.5″W) from August to December 2013 (first season), and from August to December 2014 (second season). Full bloom was on August 30, 2013, for the first season and on August 13, 2014, for the second season. For the first season, thinning was performed at 42 days after bloom (DAB), while for the second season, it was performed at 63 DAB. The thinning treatments comprised unthinned trees (UTH) that consisted in maintaining the whole fruit load resulting in a final ratio of six leaves per fruit and thinned trees (TH) that consisted in removing the fruit to the proportion 40 leaves per fruit, which is used in commercial orchard. Considering that branches of *P. persica* are autonomous concerning carbon assimilation (Volpe et al., 2008; Andrade et al., 2019), the branches were considered as biological replicates in each tree (with three trees per treatment in each year). Samples for metabolite and phenotyping measurements were harvested using four branches of each tree/thinning treatment (*n* = 12) every 7 days during the whole season in a randomized block design. Fruit growth was determined by measuring the equatorial diameter of 10 fruits per treatment weekly from 5 DAB until harvest at 118 DAB in the first season and from 27 DAB until harvest at 138 DAB in the second season. Once the fruits were harvested, they were stored at 20°C for 11 days until reaching the “ready to eat” stage.

### Quality Parameters

For both seasons, nectarines were harvested based on fruit firmness values (40–50 N). Recently harvested fruits were transported to the laboratory to measure weight, diameter, TSS using a manual refractometer, firmness of two sides of the fruit using a penetrometer, and titratable acidity (using NaOH 0.1 N) of 30–36 nectarines per tree that belonged to different branches (Supplementary Table S1 and Supplementary Figure S1). The same measurements were performed at “ready to eat” nectarines apart from the juice percentage (absorbent tissue, Infante et al., 2009). The variability was evaluated by a box plot analysis (Supplementary Figure S1) and fruits with outlier values were not used (values shaded in gray in Supplementary Table S1). All the nectarines were cut, endocarp was discarded, and mesocarp was frozen in liquid nitrogen and stored at −80°C for further analysis.

### Lignin Staining

To determine the start of pit hardening, lignin staining was performed as described by Dean (1997). Fruit was sectioned in transverse and longitudinal sections and then placed immediately in phloroglucinol-HCl staining solution (5% phloroglucinol, 85% ethanol). The excess was removed and fuming HCl was added to start the reaction causing the lignin to become magenta. The samples were washed using 95% ethanol.

### RT-PCR of Fruit Development Gene Markers

A piece of frozen fruit was ground using a mortar and pestle previously chilled with liquid nitrogen, then 3 g of sample was used for the RNA extraction following the protocol of Gudenschwager et al. (2012). One microgram of RNA treated with 2 U of DNase I (Invitrogen, CA, EE.UU) was used for cDNA synthesis using the SuperScript^TM^ First-Strand Synthesis System for RT-PCR (Invitrogen). The sequences of the primers used in the qRT-PCR assays are listed in Supplementary Table S2.

### Extraction of Polar Metabolites and Measurement by 1D ^1^H-NMR

Polar metabolites were extracted from mesocarp lyophilized powder (30 mg DW) using a hot ethanol/water series and determined using proton nuclear magnetic resonance spectroscopy (^1^H-NMR) profiling as described previously (Botton et al., 2016). pH-adjusted extracts in 200 mM deuterated phosphate buffer were analyzed using a 500 MHz Avance III spectrometer (Bruker, Wissembourg, France) with a BBI 5 mm Bruker probe. The ERETIC method was used for quantification of absolute concentration of all identified metabolites with three calibration curves (glucose and fructose: 2.5, 5, 10, 25, 50, and 100 mM; quinic acid: 1, 2.5, 5, 10, and 15 mM) using Amix Bruker v. 3.9.14 software (Supplementary Data S6). The glucose calibration curve was used for the quantification of all metabolites, as a function of the number of protons of selected resonances, except fructose and quinic acid that were quantified using their own calibration curve. The content of each organic acid was expressed as g of the acid form per weight unit. The metabolite concentrations in each NMR tube and the contents in fruit were calculated using AMIX (version 3.9.14, Bruker) and Excel (Microsoft, Redmond, WA, United States) software.

### Extraction of Polar Metabolite and Measurement by GC-MS

The extraction of polar metabolites was carried out with the protocol described by Hatoum et al. (2014). Briefly, 20 mg of lyophilized mesocarp was placed in a tube containing 500 μl of cold methanol, and 20 μl of 2,910 ng/μl phenyl β-D-glucopyranoside was added as an internal standard. Tubes were then incubated at 70°C for 15 min using a shaking incubator (VorTemp^TM^ 56, Labnet, Woodbridge, NJ, United States). Tubes were centrifuged for 20 min at 14,000 *g* and then the precipitate was discarded. One hundred microliters of the supernatant was dried using a stream of nitrogen gas. For derivatization, 120 μl of methoxyamine solution (Sigma-Aldrich, St. Louis, MO, United States) and pyridine 20 mg/ml (Sigma-Aldrich) was added to the dry sample and shaken for 90 min at 30°C. To each tube, 120 μl of BSTFA [*N*-*O*-Bis(trimethylsilyl)trifluoroacetamide] (Sigma-Aldrich) was added and shaken for 30 min at 37°C. The content of each tube was transferred into a vial with a micro insert.

Metabolomic analysis was performed by gas chromatography–mass spectrometry (GC-MS). Data were obtained using the protocol described by Fuentealba et al. (2017). Briefly, 1 μl of sample was injected on the GC column of an Agilent GC-MS system (GC7890 with a 5,977 single quadrupole MS with electron impact ionization source; Agilent Technologies, Palo Alto, CA, United States). Each derivatized extract was analyzed twice; a split (1:150) method was used for the abundant compounds such as major sugars and a splitless mode for the less abundant compounds such as organic acids and amino acids. The GC column used was an HP-5-MS capillary column of 30 m length, 0.25 mm internal diameter, and 0.25 μm film thickness (Agilent Technologies). For both methods (split and splitless), the injection and interface temperatures were 220 and 280°C, respectively. Helium was used as carrier gas with a constant flow of 1 ml/min. The GC temperature program started isothermal at 50°C for 1 min (acids method) or at 120°C for 1 min (sugar method) and was then ramped at a rate of 10°C/min to 310°C where it was kept for 13 min (acid method) or to 300°C for 6 min (sugar method). The total run time was 40 min for the acid method and 25 min for the sugar method. Mass spectra in the 50–600 *m/z* range were recorded at a scanning speed of 2.66 scan cycles per second. The MS ion source and quadrupole temperatures were 230 and 150°C, respectively.

Mass Hunter Data Analysis Software (Agilent Technologies) was used to deconvolute the chromatographic peaks. Identification was performed by comparing the peak retention and mass spectra to the NIST library in the quantitative method. Raw peak area data were corrected using the actual peak area of the internal standard, the sample fresh weight, and a quality control (QC) sample representative of all samples.

### Extraction of Water-Soluble Sugars and Measurement by HPAEC-PAD

The extraction of soluble sugars was performed following the protocol described by Schmitzer et al. (2011) with modifications. Three milligrams of lyophilized powder mesocarp was gently mixed with 2 ml of Milli-Q water per 2 h at room temperature. The supernatant was filtered using 0.45 μm nylon and then diluted 1:2. Sugars were measured using the HPLC Dionex DX-500 system equipped with two CarboPac PA1 (4 mm × 250 mm) analytical columns connected in series, a CarboPac PA1 (4 mm × 50 mm) guard column, and a pulse amperometric detector. Soluble sugars were separated at a flow rate of 1.5 ml min^–1^ at 40°C. The elution protocol consisted on isocratic gradients of 100 mM NaOH for 25 min. Finally, a washing step with 20 mM NaOH for 10 min was performed. Sugar content (myo-inositol, sorbitol, fructose, glucose, and sucrose) was determined by reference to a standard curve from 10 to 200 μM.

### Statistical Analyses

Phenotypic data were analyzed using Student’s *t*-test for mean comparisons. For metabolite data, principal component analysis (PCA), partial least square regression discriminant analysis (PLS-DA), and clustering analysis with Euclidian distance were performed on the normalized data from NMR and GC-MS data using Metaboanalyst 4.0 (Chong et al., 2019). All variables were mean-centered and reduced to unit variance before PCA, PLS-DA, and clustering analysis. For the clustering analysis for the different features, Euclidean distance similarity measure and ward.D clustering algorithm were used with the hclust function in stat package using the top 24 compounds filtered by one-way ANOVA.

## Results

### Characterization of Fruit Development

**FIGURE 1:**
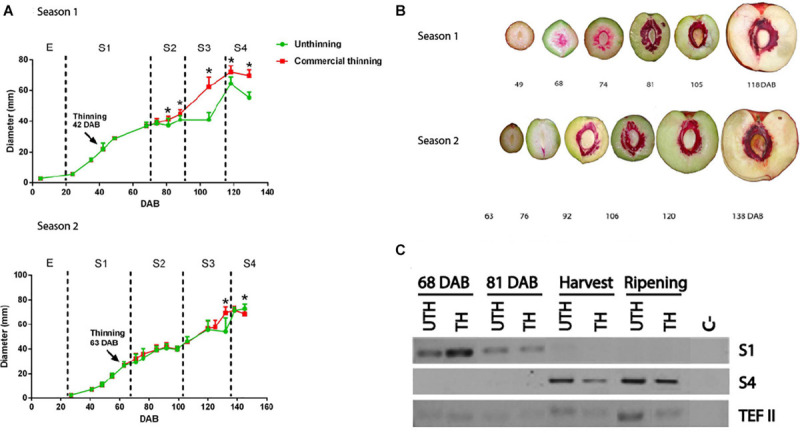
“Magique” nectarine fruit development. **(A)** Fruit growth curve. Equatorial diameter was measured throughout development of fruits during two seasons. Thinning practice was done at 42 DAB in the first season (top) and at 63 DAB during the second season (bottom) at stage 1 (S1). Green lines represent fruits from unthinned (UTH) trees and red lines from thinned (TH) trees. Each point represents the mean of 10 fruits and the bars represent SD. Asterisks denote statistical difference of fruit diameter between UTH and TH trees (Tukey test, *P* > 0.05). **(B)** Progression of lignin deposition in developing nectarine fruit. Sectioned fruits were stained with phloroglucinol-HCl for lignin staining (pink color). **(C)** Development stage gene marker for S1 (RD22-like gene) and S4 (Aux/IAA gene) measured by RT-PCR in mesocarp of nectarines from season 1 in four stages of development using fruits from UTH and TH trees. The reference gene was Translation elongation factor 2 (TEF II).

In this study, we evaluated two harvest seasons of nectarine cv “Magique.” On both seasons, trees subjected to standard commercial thinning treatments were evaluated. During both seasons, the pattern of fruit growth was the typical double-sigmoid curve in accordance with the behavior reported previously (Chalmers and van den Ende, 1975; Tonutti et al., 1991). Fruits continuously grew in an exponential rate starting at 20 DAB. This first stage of development (S1) was characterized by a first increase of fruit size, which in the first season lasted until 68 DAB, and in the second season until 70 DAB (Figure 1A). The end of S1 stage was determined by the beginning of lignin production in the endocarp (pink staining, Figure 1B). These results were consistent with the transcript expression of dehydration-responsive protein RD22 (RD22-like protein, Figure 1C), a marker gene that is expressed in fruit mesocarp mainly at this stage; this transcript is under control of ABA and seems to be related to stress response (Bonghi et al., 2011). Thinning treatment was performed during S1 stage in both seasons. In the first season, thinning was performed earlier than the second season: 42 DAB compared to 63 DAB.

Stage 2 (S2) of fruit development was characterized by pit hardening and little or no increase in fruit size, especially in early to mid-harvest nectarines such as “Magique” variety. End of S1 and start of S2 were determined based on the beginning of stone lignification (lignin becomes pink using phloroglucinol as stained in Figure 1B). During the first season, S2 lasted from 68 DAB to 88 DAB (Figures 1A,B), and in the second season, it lasted from 70 DAB to 100 DAB. In this stage, we observed the first differences in fruit size between thinned and unthinned trees in the first season, when fruits from thinned trees started to become significantly larger (Tukey test, *P* < 0.05) than fruits from unthinned trees. This trend was maintained until the end of fruit development.

In stage 3 (S3), a second increase of fruit size occurred, which corresponded to the period of 90 DAB until harvest at 118 DAB for the first season, and of 100 DAB until harvest at 138 DAB for the second season. The larger differences in fruit diameter between the thinning treatments were observed in S3 in both seasons (Figure 1A). In the first season, the rate of fruit growth during S3 was slower in unthinned trees compared to thinned trees. Stage 4 (S4) corresponded to the postharvest period, when the fruit ripens. The harvest maturity was corroborated with the expression of auxin-responsive protein IAA gene (Aux/IAA), a marker gene for S4 (Bonghi et al., 2011; Figure 1C).

The effect of thinning treatment on final fruit size was observed in the first season only (Figure 1A) when fruits from thinned trees were considerably larger than fruits from unthinned trees.

### Thinning Treatment Modified Quality Parameters of “Magique” Nectarine Differently in the Two Seasons

To evaluate quality parameters of fruits such as firmness, weight, TSS, and titratable acidity, these parameters were measured at harvest and “ready to eat” stages in the two seasons (Table 1). In both seasons, fruits were harvested with a firmness value of around 40–47 N. After 10 days at 20°C, firmness steeply decreased. In the first season, fruits from unthinned (UTH) trees presented a firmness of 12.5 N at the ready-to-eat stage while fruits from thinned (TH) trees soften much more rapidly reaching 0.4 N at ripe stage. In the second season, the softening of UTH and TH fruits was very similar, reaching 6.4 and 8.9 N of firmness at ripe stage, respectively (Table 1). On one hand, in the first season, TH fruit weight was significantly higher than UTH fruit weight in both stages (Tukey test, *P* < 0.05). On the other hand, in the second season, no differences in fruit weight were observed between treatments (Table 1). During the first season, TH fruits displayed significantly higher TSS than UTH fruits at harvest and ripening stages. Unfortunately, only one fruit from thinning treatment was analyzed for acidity, so in this point, no statistical analysis could be performed. However, fruits from TH trees in the second season showed no differences in TSS compared to fruits from UTH trees at harvest, but when they ripened, fruit from TH trees had significantly lower TSS than fruit from UTH trees (Table 1). For acidity in the second season, no differences were found between fruits from TH or UTH trees at ripe stage.

**TABLE 1:** Maturity and physiological parameters of “Magique” nectarines at harvest and ripening (shelf-life at 20°C).

	**Harvest**				**Ripening**			
	**1st Season**		**2nd Season**		**1st Season**		**2nd Season**	
	**UTH**	**TH**	**UTH**	**TH**	**UTH**	**TH**	**UTH**	**TH**
Firmness (N)	44.69 ± 2.29*	47.51 ± 1.42*	41.76 ± 9.08	44.97 ± 2.91	12.54 ± 5.02*	0.43 ± 0.72*	6.39 ± 2.65	8.89 ± 7.35
Weight (g)	135.8 ± 21.1*	185.1 ± 37.5*	186.4 ± 20.1	192.4 ± 21.6	90.14 ± 14.1*	175.9 ± 32.8*	193.8 ± 33.4	173.0 ± 26.2
TSS (° Brix)	10.81 ± 1.23*	12.31 ± 1.49*	11.77 ± 2.40	10.97 ± 1.86	10.22 ± 1.46*	12.33 ± 1.87*	12.04 ± 1.85*	8.46 ± 1.35*
Acidity (%)	−	−	−	−	0.15	1	1.36 ± 0.19	1.61 ± 0.30

TSS, total soluble solids; UTH, unthinning; TH, thinning. *Values (means) followed by asterisks are significantly different between thinning treatments at Tukey test P < 0.05.

### Effect of Thinning Treatment on Metabolite Profiles of “Magique” Nectarines

**FIGURE 2:**
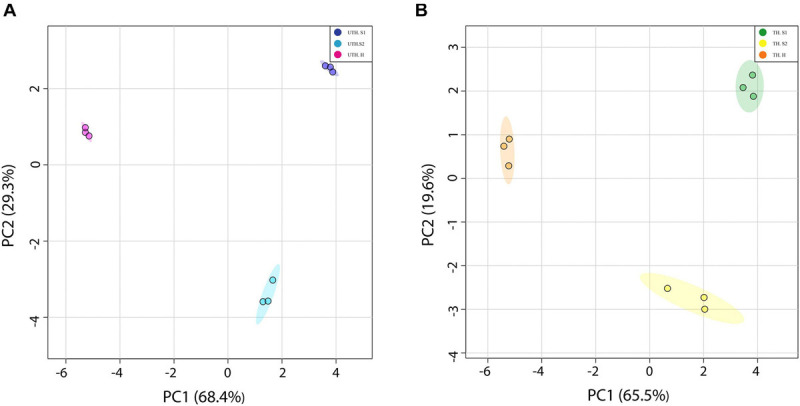
Principal component analysis (PCA) of “Magique” nectarine metabolites detected using ^1^H-NMR. The detected metabolites were employed as predictor variables, and the stages of development were used as response variables. The left panel **(A)** shows the score plot of S1, S2, and harvest (H) stages from unthinned (UTH) trees during the first season, where the explained variance by each principal component corresponded to 68.4 and 29.3% for PC1 and PC2, respectively. The right panel **(B)** shows the score plot of S1, S2, and harvest (H) stages from thinned (TH) trees during the first season, where the explained variance by each principal component corresponded to 69.5 and 19.6% for PC1 and PC2, respectively. Dark blue circles represent UTH S1, light blue circles represent UTH S2, and pink circles represent UTH harvest (H) fruit samples. Green circles represent TH S1, yellow circles denote TH S2, and orange circles represent TH H fruit samples.

Metabolite profiles (mainly primary metabolites) were determined using ^1^H-NMR metabolomics profiling in three stages of development: S1 (68 DAB), S2 (81 DAB), and harvest (118 DAB) in the first season. A total of 24 polar metabolites were quantified in all stages of development and thinning treatment (Supplementary Tables S3, S4). The data of each thinning treatment were evaluated separately by an unsupervised multivariate statistical analysis using the stage of development as response variables and the identified metabolites as the predictor variables. PCA of nectarine metabolites that belong to UTH trees was able to explain 68.4 and a 29.3% of total variance with the first two components (Figure 2A). Similarly, PCA of TH was able to explain 65.5 and 19.6% for PC1 and PC2, respectively (Figure 2B). Interestingly, both projections clearly separate by developmental stages and the distribution of groups was similar as well. PC1 tended to separate earlier stages (S1 and S2) on the positive side from harvest (H) on the negative side. In the second season, the metabolite profiles were measured by GC-MS, and no compositional differences between UTH and TH fruits were found for the 124 compounds quantified (Supplementary Table S5 and Supplementary Figure S2), in agreement with no changes in phenotypic parameters such as fruit weight.

**FIGURE 3:**
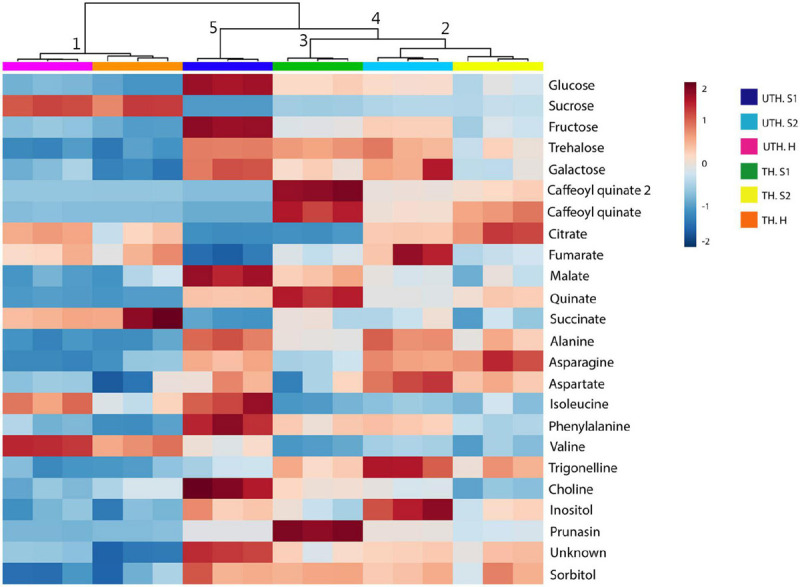
Heatmap analysis of “Magique” nectarine metabolites detected by ^1^H-NMR at S1, S2, and harvest (H) stages from unthinned (UTH) and thinned (TH) trees during the first season. The analysis was based on the top 24 significant metabolites revealed by ANOVA. The columns represent the biological replicates for each stage (S1, S2, and Harvest). Dark blue rectangles represent UTH S1, light blue rectangles represent UTH S2, and pink rectangles represent UTH harvest (H) fruit samples. Green rectangles represent TH S1, yellow rectangles denote TH S2, and orange rectangles represent TH H fruit samples. Cluster numbers are indicated on the upper side of the figure. The distance measure for the different features was Euclidean and the clustering algorithm corresponded to Ward.

In order to determine how thinning treatment affected metabolite profile, a clustering analysis was performed based on the top 24 metabolites shown by ANOVA in the first season and visualized with a heatmap (Figure 3). Harvest samples (H) of both thinning treatment grouped together in cluster 1. Cluster 2 grouped S2 of both UTH and TH, and shared node with cluster 3 that grouped only S1 TH. S1 UTH clustered separately from S1 TH. The main differences in accumulation of metabolites in nectarine samples between thinning treatments were found in S1, in a lesser extent in S2, and a few differences in H. For example, caffeoyl quinate, caffeoyl quinate 2, and prunasin were completely absent in S1 UTH, while in S1, TH were highly abundant. On the other hand, several metabolites were more accumulated in S1 UTH than S1 TH such as glucose, fructose, malate, isoleucine, phenylalanine, and choline. In S2, the main differences were found in galactose, fumarate, and inositol, which were more abundant in UTH than TH.

**FIGURE 4:**
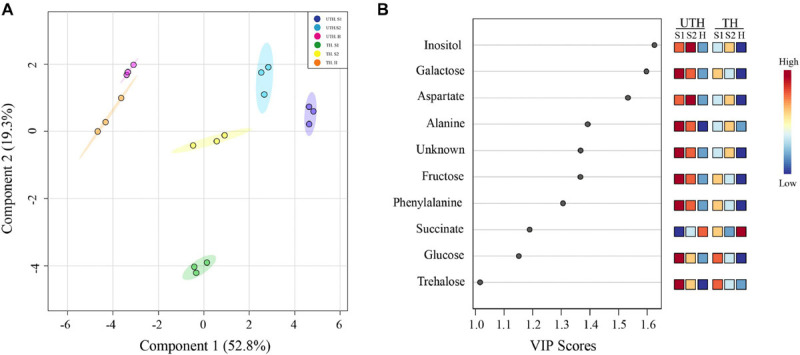
Partial least square discriminant analysis (PLS-DA) of “Magique” nectarine metabolites detected using ^1^H-NMR for first season. The detected metabolites were employed as predictor variables, and the stages of development and thinning treatments were used as response variables. The left panel **(A)** shows the loading plot where the explained variance by each component corresponded to 52.8 and 19.3% for C1 and C2, respectively. Dark blue circles represent UTH S1, light blue circles represent UTH S2, and pink circles represent UTH harvest (H) fruit samples. Green circles represent TH S1, yellow circles represent TH S2, and orange circles represent TH H fruit samples. The right panel **(B)** shows the top 10 variable importance in projection (VIP) identified by PLS-DA. The colored boxes on the right indicate the relative concentrations of each metabolite at each stage of development.

A multivariate statistical analysis was performed with the data obtained from both UTH and TH samples, using detected metabolites as predictor variables and stage of development and thinning treatments as response variables. PLS-DA was able to explain 52.8 and 19.3% of total variance with the two components (Figure 4A). In this projection, C2 tended to separate by thinning treatments, where in the positive side was UTH and in the negative side was TH, except for harvest from TH (TH H), which is in the middle tending to the positive side. C1 separated the early stages of development from harvest independently of thinning treatment. On the positive side of C1 were UTH S1 and S3, as well as TH S3. TH S1 metabolite composition was very different from all other samples. TH S1 was at the negative side of C2, isolated from all the others. At this same position, caffeoyl quinate 1, caffeoyl quinate 2, prunasin, and quinate were observed in the loadings plot (Supplementary Figure S3). The 10 most important metabolites involved in the discrimination between developmental stages and thinning treatment were obtained by a variable importance in projection (VIP) analysis identified by PLS-DA (Figure 4B). Inositol, galactose, aspartate, alanine, unknown (multiplet at 0.97 ppm) compound, fructose, phenylalanine, succinate, glucose, and trehalose were the most important metabolites identified by PLS-DA.

**FIGURE 5:**
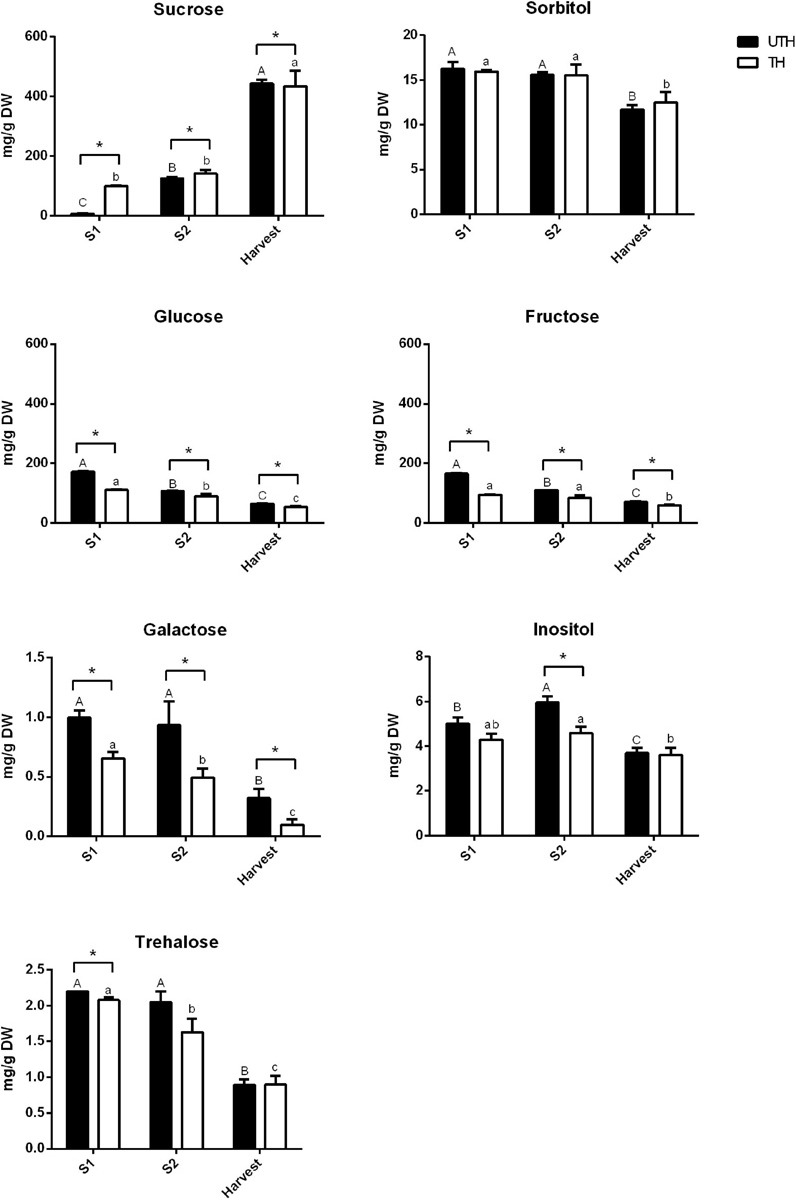
Contents of sugars in fruits from unthinned (UTH) and thinned (TH) trees during first season development. Each column represents the average of three biological replicates (*n* = 3); bars are the standard deviations. Capital letters indicate comparison of each sugar concentration during UTH fruit development. Lowercase letters indicate comparison of each sugar concentration during TH fruit development. Asterisks indicate differences between thinning treatments. Statistical analysis was performed by Holm–Bonferroni method using *P* < 0.05.

**FIGURE 6:**
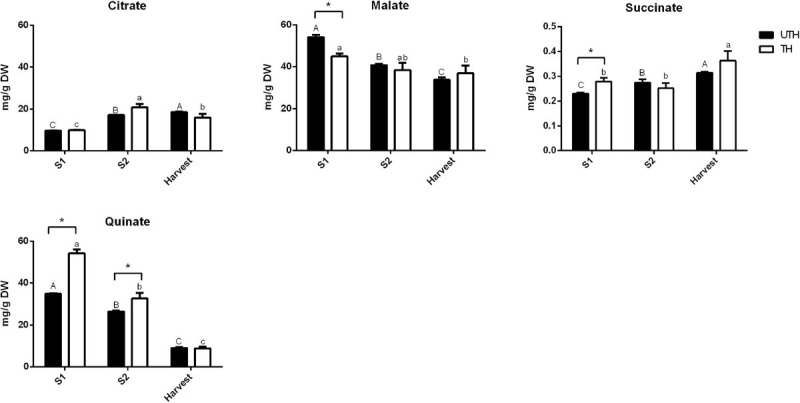
Contents of organic acids in fruits from unthinned (UTH) and thinned (TH) trees during first season development. Each column represents the average of three biological replicates (*n* = 3); bars are the standard deviations. Capital letters indicate comparison of each organic acid concentration during UTH fruit development. Lowercase letters indicate comparison of each organic acid concentration during TH fruit development. Asterisks indicate differences between thinning treatments. Statistical analysis was performed by Holm–Bonferroni method using *P* < 0.05.

**FIGURE 7:**
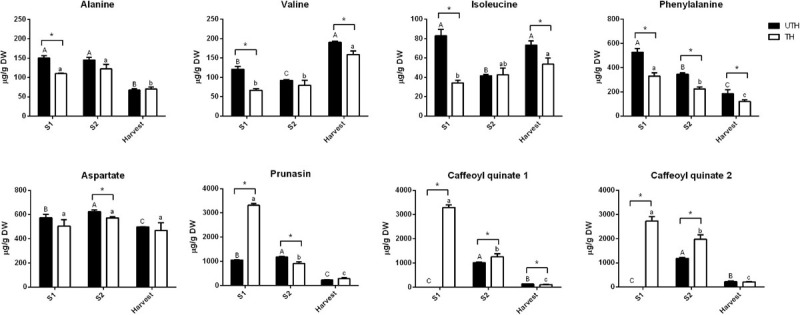
Contents of amino acids and phenylpropanoid pathway intermediates in fruits from unthinned (UTH) and thinned (TH) trees during first season development. Each column represents the average of three biological replicates (*n* = 3); bars are the standard deviations. Capital letters indicate comparison of each metabolite concentration during UTH fruit development. Lowercase letters indicate comparison of each metabolite concentration during TH fruit development. Asterisks indicate differences between thinning treatments. Statistical analysis was performed by Holm–Bonferroni method using *P* < 0.05.

Figures 5, 6 show the changes of the main soluble sugars and organic acids during fruit development in the first season. As expected, sucrose was the predominant sugar at harvest in both UTH and TH fruits (Figure 5) and its concentration increased during fruit development. Sorbitol and trehalose remained practically constant during development, showing just a decrease in harvest stage in both thinning treatments. Hexose concentrations decreased during fruit development. The most striking differences observed between thinning treatments were that sucrose concentration was significantly higher in TH fruit, while hexose concentrations were significantly and consistently higher in UTH fruit in all development stages, with the exception of H where sucrose was similar in both treatments. Inositol, the metabolite that presented the highest VIP, had its concentration discretely increased in S2 and then diminished at harvest. However, during S2, its concentration was different between thinning treatment. Soluble sugars from mesocarp of fruits at harvest and ripening stages were also measured in parallel using HPAEC-PAD. Myo-inositol, sorbitol, glucose, fructose, and sucrose were found in similar proportions in both thinning treatments at the harvest stage (Supplementary Figure S4). In order to compare the results obtained by this method, the data obtained by NMR were plotted as proportions. A similar trend was found where sucrose was the most predominant sugar at the harvest stage, being more abundant in TH than UTH fruits (Supplementary Figure S4). Sorbitol was found in less concentration and inositol was found as just traces. At the ripening stage, sucrose proportion increased compared to the other sugars measured and reached approximately 89% of total sugar in TH reached and 75.5% in UTH (Supplementary Figure S4). Concerning organic acids, differences between thinning treatments were observed in the earlier stages of development (Figure 6). Succinate concentration was significantly higher in TH than in UTH in S1, but the differences disappeared along development, gradually increasing its concentration similar to citrate but in a different scale. Malate concentration was significantly higher in UTH than in TH S1 fruit and decreased along development. On the other hand, quinate concentration was significantly higher in TH than in UTH fruit at S1 and S2 developmental stages and also decreased until harvest. Phenylalanine content (Figure 7) was higher at S1 phase in both UTH and TH fruits and then decreased smoothly in S2 and harvest stages, and its content was significantly higher in UTH fruits than in TH fruits at all stages. Similarly, alanine concentration decreased along the development, but from S2 to harvest, the decrease was steep. Alanine content was higher in UTH than in TH in S1, while in the other stages of development, there was no difference between thinning treatments. Isoleucine and valine showed the same pattern with a decrease from S1 until S2 and then an increase at harvest in the UTH fruit (Figure 7). On the other hand, these amino acids showed constant concentrations in TH fruit during development. The amount of these amino acids was higher in UTH than TH fruit. Aspartate evolution showed a different pattern. This amino acid was the most accumulated throughout all fruit development in both thinning treatments followed by phenylalanine (Figure 7). Aspartate concentration remained practically steady and in equivalent concentrations in both thinning treatments. The only exceptions were at S2 when UTH fruits presented more aspartate than TH. The two caffeoyl quinates and prunasin showed the same concentration pattern in both thinning treatments throughout fruit development (Figure 7) with higher contents at S1 and a steep decrease until harvest. These compounds were consistently higher in TH than in UTH fruit.

## Discussion

Thinning is an agronomical practice used worldwide in several fruit tree species to increase fruit size (Myers et al., 2002). It is very well described in the literature that thinning affects fruit size and even development time. It is also known that thinning may impact organoleptic traits such as TSS content, without any detail of the metabolite composition changes induced by this agronomical practice (Costa et al., 2018). The effectiveness of thinning depends largely on the stage of development in which it is performed. The recommendation is to perform thinning at the beginning of fruit development before the mesocarp sucrose accumulation starts (Costa and Vizzotto, 2000). In this study, we analyzed the mesocarp metabolic profile during the development of nectarine fruits when trees were subjected to different thinning treatments. We worked with an early to mid-season variety that takes around 120 DAB to be harvested in two consecutive seasons (Ognjanov et al., 1995; Figure 1). We observed an increase in final fruit size and TSS in response to thinning only in the first season evaluated (Figure 1A and Table 1). No differences in diameter, TSS, or acidity were observed between fruits from thinned compared to unthinned trees during the second season (Figure 1A and Table 1). Fruits from thinned trees seemed to soften faster than unthinned trees as after 10 days at 20°C, the firmness decreased more intensively. These differences between the seasons are probably due to the different times of thinning. In the first season, thinning was performed at 42 DAB at the middle of the first exponential growth phase (S1), while in the second season, thinning was performed at 63 DAB, which corresponds to the end of S1. During S1, the first increase in fruit volume occurs mainly due to the high rate of cell proliferation and expansion process (Scorza et al., 1991; Zanchin et al., 1994), which leads to an increase in fruit metabolic rate (Moing et al., 1998). The increase in photoassimilate demand directly impacts the strength of the fruit as a sink (Grossman and DeJong, 1995). Unfortunately, we did not count the number of mesocarp cells in the fruit to verify this hypothesis. It was shown that peach trees with light to moderate crop load (thinned trees) harbor fruit with higher dry matter and water potential (Berman and DeJong, 1996). On the other hand, a strong cutinization of the epidermal layer occurs during S3 that might be related to limited respiration to avoid water loss (Masia et al., 1992). When crop load was high (unthinned trees), photoassimilates had to be distributed among many fruits, resulting in smaller fruit with lower dry matter. This limitation in the growth rate due to limited photoassimilate resources is called source-limited (Pavel and DeJong, 1993). The S1 stage is the first source-limited growth period. During the first harvest season, thinning was performed at the beginning of S1 phase diminishing the competition of resources among the remaining fruit. In the second season, thinning was performed late during S1, after the first source-limited growth period (the second was during S3 stage), and the remaining fruits were unable to take full advantage of the decreased competition for resources caused by thinning and potential yield was lost (Grossman and DeJong, 1995). In other Prunus species, such as sweet cherry (*Prunus avium*) and plum (*P. salicina, P. domestica*, and *P. cerasifera*), it was shown that genotypic differences in final fruit size are primarily a function of cell number (Olmstead and Iezzoni, 2007; Cerri et al., 2019). It is possible that the lower competition for photoassimilates in trees that were thinned at the beginning of S1 phase (first season) stimulated a higher post-bloom cell division rate compared to the unthinned condition, positively impacting the final fruit size.

The metabolic profile of peach fruit has been broadly studied at different stages of development, during postharvest and treatments to prevent chilling injury (Moing et al., 1998; Lombardo et al., 2011; Botton et al., 2016; Brizzolara et al., 2018; Nuñez et al., 2019; Lillo-Carmona et al., 2020). Here, we showed the metabolic response to a source–sink manipulation removing fruits in order to obtain an approximately 36 leaves per one fruit in a branch (thinning performed in commercial orchards in Chile). A total of 24 metabolites were quantified by ^1^H-NMR profiling: 7 sugars, 7 organic acids, 6 amino acids, 2 amine derivatives, 1 cyanogenic glucoside, and 1 unknown compound. PCA analysis revealed that each stage of development has their unique metabolic profile since each group was separated from the others in both thinning treatments (Figure 2). On the other hand, the heatmap analysis showed that S1 showed a different metabolic profile depending on the thinning treatment (Figure 3), and along the development of the nectarines, these differences decrease. The S1 samples used for metabolite analysis were sampled 26 days after thinning, sufficient time to observe any effect of the treatment. Since the main compositional separation was observed during earlier stages, we suggest that an early change of the metabolite profile could witness metabolism rearrangements that cause changes in the final stages of development.

We had shown before that sorbitol was the most abundant soluble sugar in “Magique” leaves followed by sucrose and that thinning induced an increase in the sugar contents in leaves at S1 and S2 phases (Andrade et al., 2019). These are the main exportable sugars in *P. persica*, and they were previously reported as the major soluble sugars in the leaves of this species (Moing et al., 1992). The increased content of exportable sugars together with the increase in the expression of sugar transporters related to phloem loading/unloading in response to thinning (Nuñez et al., 2019; Aslani et al., 2020) probably sustained the high metabolic rates and carbohydrate demand to sustain cell proliferation and endocarp lignification at S1 and S2 phases in fruit from thinned trees. We did not observe accumulation of these sugars in fruits, because once they arrived in the fruit, they were converted to glucose and fructose to sustain respiration and also directed to other biosynthetic pathways such as synthesis of cell wall components, amino acids, and precursors related to lignin synthesis. The higher content of hexoses in S1 UTH fruits in comparison to TH fruits may be due to the lower metabolic rates of UTH fruit. Inositol is a polyol that maintained low levels throughout the development with a peak at S2. This metabolite was the highest VIP, indicating its importance in the separation of the variables in PLS-DA. It also showed a strong correlation with early stages of UTH fruit development (Supplementary Figure S3). Inositol is synthesized from glucose 6-phosphate and can act in different plant metabolisms as cell wall biosynthesis, auxin physiology, and response to stress among others (Loewus and Murthy, 2000). At the harvest stage, inositol contents are equivalent in UTH and TH. Its accumulation in S2 UTH could suggest a delay in the development of UTH fruits.

Previous studies showed that lignin gene expression is induced at extremely high levels just before the end of S1 phase (Dardick et al., 2010). Phenylalanine is an aromatic amino acid and the main phenylpropanoid pathway precursor that leads to lignin and flavonoid biosynthesis (Singh et al., 2010; Botton et al., 2016). Our data showed that this amino acid accumulated at S1 phase in both UTH and TH fruit, followed by its content decreasing in S2 and S3 phases. Nevertheless, phenylalanine content was higher in S1 UTH fruits than TH fruits (Figures 3, 7). The same pattern was observed for alanine, asparagine, isoleucine, and valine (Figure 7), which are also substrates for the phenylpropanoid pathway that lead to lignin and flavonoid biosynthesis (Lombardo et al., 2011). In contrast, quinate and caffeoyl quinate 1 and 2, which belong to this same pathway downstream phenylalanine (Del Cueto et al., 2018), were more abundant in S1 TH than UTH (Figures 6, 7). These metabolites and prunasin showed a high correlation with S1 UTH samples (Supplementary Figure S3). Thinning may also advance fruit development and harvest (Nuñez et al., 2019). It seems that UTH fruits were delayed in contrast to TH fruits, because the content of phenylalanine during S2 UTH was similar to S1 TH. Therefore, phenylalanine previously accumulated in TH fruit was already used for lignin synthesis and endocarp hardening. Indeed, prunasin is a cyanogenic glucoside biosynthesized from phenylalanine and then metabolized into amygdalin, which is localized only in seeds at later developmental stages (Mizutani et al., 1991). It was detected in higher amounts in TH S1 than UTH S1 fruit, indicating that endocarp development was advanced in TH fruit. This is in agreement with the UTH fruit growth curve shown in Figure 1A, where S2 lasted more in UTH than TH. The pit hardening process requires high amounts of energy sustained by imported photoassimilates (Dardick et al., 2010), which were more abundant in TH than in UTH trees. This increased availability of photoassimilates in TH trees accelerated fruit development. Aspartate, the third metabolite by VIP, is involved in the biosynthesis of asparagine, lysine, threonine, isoleucine, and methionine (Azevedo et al., 2005). Besides, its concentration was almost constant throughout the development, except for S2 UTH (Figure 7), which tended to accumulate, suggesting that the metabolism of aspartate was slower than in TH fruit, in agreement with the hypothesis that UTH fruits were delayed in development.

All the sugars detected had an accumulation pattern characterized by higher contents at early stages and progressively declined until harvest. The only exception was sucrose that presented exactly the opposite trend, with lower levels at early stages of development and being the predominant sugar at late stages. In the fruit, high hexose concentrations observed in S1 were associated to high sucrose synthase (SS, EC 2.4.1.13) and acid invertase (AI, EC 3.2.1.26) activities (Moriguchi et al., 1990; Lo Bianco and Rieger, 2002) when the energy requirement of the dividing cells was high. Trehalose evolution during development presented exactly the opposite trend of sucrose independent of thinning treatment (Figure 5). Trehalose is the precursor of trehalose 6-phosphate (Tre6P), which is a critical signaling metabolite that is important for plant growth and development at all stages of the plant’s life cycle (Figueroa and Lunn, 2016). In sink organs where sucrose and sorbitol in Rosaceae species are the major carbon import, it has been suggested that Tre6P promotes growth and acts as a major hub when sucrose supply is high (Schluepmann et al., 2003; Baena-González and Lunn, 2020). The decreased trehalose concentration may reflect an increase in Tre6P production signaling sucrose availability at the different stages of fruit development.

The main organic acids found in all stages of development of “Magique” nectarine were malate and quinate (Figure 6) whose content declined along development. On the other hand, succinate, citrate, and fumarate were found in a lesser extent, and their content rose to reach a maximum at harvest (Figures 3, 6). This profile was similar to the high-acid peach cultivars (Wu et al., 2005; Zheng et al., 2021). Taste is a multifactorial aspect in which sweetness and sourness play a key role. Sugars and organic acids are involved in both characteristics, respectively (Cirilli et al., 2016). Sucrose accumulation at the end of peach development originates from phloem import and gluconeogenesis with organic acids as substrates and is directly related to the fruit sweetness (Vizzotto et al., 1996; Cirilli et al., 2016; Desnoues et al., 2016). Sucrose presented its highest content at harvest. NMR was used to measure metabolites in three stages of development, from S1 until harvest while HPLC was used to measure water-soluble sugars at harvest and ripening stages. NMR and HPLC data were quite similar at the harvest stage when a higher proportion of sucrose was observed in TH than in UTH fruits (Supplementary Figure S4). This trend was exacerbated at the ripening stage in agreement with phenotypic analysis, where TSS of TH were higher than UTH fruits in the first season evaluated (Table 1). This pattern of sugar accumulation throughout fruit development in “Magique” is quite similar to that observed in other peach and nectarine varieties (Moing et al., 1998; Lombardo et al., 2011; Zhang et al., 2013; Bae et al., 2014; Desnoues et al., 2014; Roch et al., 2020). Nuñez et al. (2019) reported that this steep increase of sucrose at the end of development was in parallel to an increase in the sucrose transporter gene *PpeSUT1* expression mainly in thinned trees probably supporting apoplasmic sucrose unloading at harvest. We also observed higher sucrose content at ripening in TH fruits compared to UTH (Supplementary Figure S4), which sustains this hypothesis. The organic acid accumulation during early stages of fruit development is directly related to the supply of substrates for respiration process maintenance during fruit development (Seymour et al., 2013) while its decrease at later stages is related to their consumption for sucrose synthesis.

## Conclusion

Our results indicated that fruit thinning in peach (i.e., source–sink imbalance) should be performed at the beginning of fruit development to be effective in increasing final fruit size. We showed that when performed at the right moment, thinning affects the metabolite composition of fruit mainly in earlier stages of development and driving resources to sustain cell division that will impact final fruit size. Metabolite data revealed that sugar, organic acid, and phenylpropanoid pathways at early stages of development (S1 and S2 stages) can be used to segregate fruits impacted by the change in source–sink balance. In conclusion, we suggest that the profile of these metabolites in early developmental stages could be a metabolite predictor of final fruit quality in nectarines.

## Data Availability Statement

The original contributions presented in the study are included in the article/Supplementary Material, further inquiries can be directed to the corresponding author/s.

## Author Contributions

AMA and MLV designed the research. MPC, LM, GB, and DA conducted field work, fruit phenotyping, and sampling. MPC, MM, CD, and AM performed proton NMR metabolite analyses. VL-C, CF, and RP performed GC-MS analyses and data analysis using Metaboanalyst 4.0. MPC, LM, GB, and MLV performed HPAEC-PAD analyses. MC and AMA wrote the original manuscript. MPC, VL-C, AM, RP, and AMA edited and reviewed the original manuscript. All authors read and approved the submitted version.

## Conflict of Interest

The authors declare that the research was conducted in the absence of any commercial or financial relationships that could be construed as a potential conflict of interest.

## Supplementary Material

Supplementary Figure 1 Phenotypic analysis of “Magique” nectarines harvested from unthined (UTH) and thinned (TH) trees in both seasons. A boxplot analysis was performed to select the most similar fruits. In harvest, firmness was evaluated and in ripening firmness and juiciness were analyzed.

Supplementary Figure 2 Principal component analysis (PCA) of the 124 metabolites detected by GC-MS at S1, S3 and harvest (H) stage from unthinned (UTH) and thinned (TH) trees during the second season. The detected metabolites were employed as predictor variables, and the stages of development as a response variable. The panel shows the score plot where the variance explained of each component corresponded to 21.1% for PC1 and 13.7% for PC2. Blue circles represent S1 fruit samples from unthinned trees and green circles from thinned trees. Light blue circles represent S3 fruit samples from unthinned trees and yellow circles from unthinned trees. Pink circles represent harvested fruit samples from unthinned trees and orange circles from thinned trees.

Supplementary Figure 3 Loadings plot between the selected PCs of Partial Least Square Analysis Discriminant Analysis (PLS-DA) of “Magique” nectarine metabolites detected using ^1^H-NMR for first season.

Supplementary Figure 4 Sugar proportions in the mesocarp of “Magique” nectarine fruit during the first season measured by HPAEC-PAD (harvest and ripening stages) and ^1^H-NMR (harvest stage). The total concentration of myo-inositol, sorbitol, glucose, fructose and sucrose together was considered as 100% and the proportion of each sugar was plotted.

Supplementary Table 1 Phenotypic data obtained from fruit harvested in both seasons evaluated.

Supplementary Table 2 Primers used for RT PCR analysis.

Supplementary Table 3 Metabolites quantified by ^1^NMR in the mesocarp of nectarines at different stages of development in the first season.

Supplementary Table 4 ^1^H chemical shifts (δ_H_ in ppm) of caffeoyl quinic derivatives.

Supplementary Table 5 Metabolites quantified by GC-MS in the mesocarp of nectarines at different stages of development in the first season.

Supplementary Data 6 NMR calibration curves.

## References

[B1] Andrade D. Covarrubias M. P. Benedetto G. Pereira E. G. Almeida A. M. (2019). Differential source–sink manipulation affects leaf carbohydrate and photosynthesis of early- and late-harvest nectarine varieties. *Theor. Exp. Plant Phys.* 31 341–356. 10.1007/s40626-019-00150-0

[B2] Aslani L. Gholami M. Mobli M. Ehsanzadeh P. Bertin N. (2020). Decreased sink/source ratio enhances hexose transport in the fruits of greenhouse tomatoes: integration of gene expression and biochemical analyses. *Physiol. Plant.* 170 120–131. 10.1111/ppl.13116 32356387

[B3] Azevedo R. A. Lancien M. Lea P. J. (2005). The aspartic acid metabolic pathway, an exciting and essential pathway in plants. *Amino Acids* 30 143–162. 10.1007/s00726-005-0245-2 16525757

[B4] Bae H. Yun S. K. Jun J. H. Yoon I. K. Nam E. Y. Kwon J. H. (2014). Assessment of organic acid and sugar composition in apricot, plumcot, plum, and peach during fruit development. *J. Appl. Bot. Food Qual.* 87 24–29. 10.5073/JABFQ.2014.087.004

[B5] Baena-González E. Lunn J. E. (2020). SnRK1 and trehalose 6-phosphate – two ancient pathways converge to regulate plant metabolism and growth. *Curr. Opin. Plant Biol.* 55 52–59. 10.1016/j.pbi.2020.01.010 32259743

[B6] Berman M. E. DeJong T. M. (1996). Crop load and water stress effects on daily stem growth in peach (*Prunus persica*). *Tree Physiol.* 17 467–472. 10.1093/treephys/17.7.467 14759839

[B7] Bonghi C. Trainotti L. Botton A. Tadiello A. Rasori A. Ziliotto F. (2011). A microarray approach to identify genes involved in seed-pericarp cross-talk and development in peach. *BMC Plant Biol.* 11:107. 10.1186/1471-2229-11-107 21679395 PMC3141638

[B8] Botton A. Rasori A. Ziliotto F. Moing A. Maucourt M. Bernillon S. (2016). The peach HECATE3-like gene FLESHY plays a double role during fruit development. *Plant Mol. Biol.* 91 97–114. 10.1007/s11103-016-0445-z 26846510

[B9] Brasil I. M. Siddiqui M. W. (2018). “Postharvest quality of fruits and vegetables: an overview,” in *Preharvest Modulation of Postharvest Fruit and Vegetable Quality*, ed. Siddiqui M. W. (Cambridge, MA: Academic Press), 1–40. 10.1016/B978-0-12-809807-3.00001-9

[B10] Brizzolara S. Hertog M. Tosetti R. Nicolai B. Tonutti P. (2018). Metabolic responses to low temperature of three peach fruit cultivars differently sensitive to cold storage. *Front. Plant Sci.* 9:706. 10.3389/fpls.2018.00706 29892309 PMC5985494

[B11] Byers R. E. Marini R. (1994). Influence of blossom and fruit thinning on peach flower bud tolerance to an early spring freeze. *HortScience* 29 146–148. 10.21273/hortsci.29.3.146

[B12] Carrari F. Fernie A. R. (2006). Metabolic regulation underlying tomato fruit development. *J. Exp. Bot.* 57 1883–1897. 10.1093/jxb/erj020 16449380

[B13] Cerri M. Rosati A. Famiani F. Reale L. (2019). Fruit size in different plum species (genus *Prunus* L.) is determined by postbloom developmental processes and not by ovary characteristics at anthesis. *Sci. Hortic.* 255 1–7. 10.1016/j.scienta.2019.04.064

[B14] Chalmers J. D. van den Ende B. (1975). A reappraisal of the growth and development of peach fruit. *Aust. J. Plant Physiol.* 2 623–634. 10.1071/PP9750623

[B15] Cherian S. Figueroa C. R. Nair H. (2014). ‘Movers and shakers’ in the regulation of fruit ripening: a cross-dissection of climacteric versus non- climacteric fruit. *J. Exp. Bot.* 65 4705–4722. 10.1093/jxb/eru280 24994760

[B16] Chong J. Wishart D. S. Xia J. (2019). Using metaboanalyst 4.0 for comprehensive and integrative metabolomics data analysis. *Curr. Protoc. Bioinformatics* 68:e86. 10.1002/cpbi.86 31756036

[B17] Cirilli M. Bassi D. Ciacciulli A. (2016). Sugars in peach fruit: a breeding perspective. *Hortic. Res.* 3:15067. 10.1038/hortres.2015.67 26816618 PMC4720000

[B18] Colaric M. Veberic R. Stampar F. Hudina M. (2005). Evaluation of peach and nectarine fruit quality and correlations between sensory and chemical attributes. *J. Sci. Food Agric.* 85 2611–2616. 10.1002/jsfa.2316

[B19] Costa G. Botton A. Vizzotto G. (2018). Fruit thinning: advances and trends. *Hortic. Rev.* 46 185–226. 10.1002/9781119521082.ch4

[B20] Costa G. Vizzotto G. (2000). Fruit thinning of peach trees. *Plant Growth Regul.* 31 113–119. 10.1023/a:1006387605042

[B21] Dardick C. D. Callahan A. M. (2014). Evolution of the fruit endocarp:molecular mechanisms underlying adaptations in seed protection and dispersal strategies. *Front. Plant Sci.* 5:284. 10.3389/fpls.2014.00284 25009543 PMC4070412

[B22] Dardick C. D. Callahan A. M. Chiozzotto R. Schaffer R. J. Piagnani M. C. Scorza R. (2010). Stone formation in peach fruit exhibits spatial coordination of the lignin and flavonoid pathways and similarity to *Arabidopsis* dehiscence. *BMC Biol.* 8:13. 10.1186/1741-7007-8-13 20144217 PMC2830173

[B23] Dean J. F. D. (1997). “Lignin analysis,” in *Methods in Plant Biochemistry and Molecular Biology*, ed. Dashek W. V. (Boca Raton, FL: CRC Press), 199–215.

[B24] Del Cueto J. Moller B. L. Dicenta F. Sánchez-Pérez R. (2018). β-Glucosidase activity in almond seeds. *Plant Physiol. Biochem.* 126 163–172. 10.1016/j.plaphy.2017.12.028 29524803

[B25] Desnoues E. Baldazzi V. Génard M. Mauroux J.-B. Lambert P. Confolent C. (2016). Dynamic QTLs for sugars and enzyme activities provide an overview of genetic control of sugar metabolism during peach fruit development. *J. Exp. Bot.* 67 3419–3431. 10.1186/s12870-014-0336-x 27117339 PMC4892732

[B26] Desnoues E. Gibon Y. Baldazzi V. Signoret V. Génard M. Quilot-Turion B. (2014). Profiling sugar metabolism during fruit development in a peach progeny with different fructose-to-glucose ratios. *BMC Plant Biol.* 14:336. 10.1186/s12870-014-0336-x PMC4247632 25421154

[B27] Figueroa C. M. Lunn J. E. (2016). A tale of two sugars: trehalose 6-phosphate and sucrose. *Plant Physiol.* 172 7–27. 10.1104/pp.16.00417 27482078 PMC5074632

[B28] Food and Agriculture Organization of the United Nations (FAOSTAT) (2018). Available online at: http://www.fao.org/faostat/en/#home (accessed August 19, 2020).

[B29] Fuentealba C. Hernández I. Saa S. Toledo L. Burdiles P. Chirinos R. (2017). Color and in vitro quality attributes of walnuts from different growing conditions correlate with key precursors of primary and secondary metabolism. *Food Chem.* 232 664–672. 10.1016/j.foodchem.2017.04.029 28490125

[B30] Génard M. Lescourret F. Gomez L. Habib R. (2003). Changes in fruit sugar concentrations in response to assimilate supply, metabolism and dilution: a modeling approach applied to peach fruit (*Prunus persica*). *Tree Physiol.* 23 373–385. 10.1093/treephys/23.6.373 12642239

[B31] Goff S. A. Klee H. J. (2006). Plant volatile compounds: sensory cues for health and nutritional value? *Science* 311 815–819. 10.1126/science.1112614 16469919

[B32] Grossman Y. L. DeJong T. M. (1995). Maximum fruit growth potential and seasonal patterns of resource dynamics during peach growth. *Ann. Bot.* 75 553–560. 10.1006/anbo.1995.1058

[B33] Gudenschwager O. González-Agüero M. Defilippi B. (2012). A general method for high-quality RNA isolation from metabolite-rich fruits. *S. Afr. J. Bot.* 83 186–192. 10.1016/j.sajb.2012.08.004

[B34] Hatoum D. Annaratone C. Hertog M. L. A. T. M. Geeraerd A. H. Nicolai B. M. (2014). Targeted metabolomics study of ‘Braeburn’ apples during long-term storage. *Postharvest Biol.Technol.* 96 33–41. 10.1016/j.postharvbio.2014.05.004

[B35] Infante R. Meneses C. Rubio P. Seibert E. (2009). Quantitative determination of flesh mealiness in peach [*Prunus persica* L. (Batch.)] through paper absorption of free juice. *Postharvest Biol. Technol.* 51 118–121. 10.1016/j.postharvbio.2008.06.006

[B36] Kader A. A. (2008). Perspective flavor quality of fruits and vegetables. *J. Sci. Food Agric.* 88 1863–1868.

[B37] Karlova R. Chapman N. David K. Angenent G. C. Seymour G. B. de Maagd R. A. (2014). Transcriptional control of fleshy fruit development and ripening. *J. Exp. Bot.* 65 4527–4541. 10.1093/jxb/eru316 25080453

[B38] Lesičar J. Šindrak Z. Šic J. Voca S. Skendrovic M. (2016). Influence of fruit thinning and summer pruning on the yield and fruit quality of peach cultivar ‘Royal Gem’. *Agric. Conspec. Sci.* 86 155–159.

[B39] Lillo-Carmona V. Espinoza A. Rothkegel K. Rubilar M. Nilo-Poyanco R. Pedreschi R. (2020). Identification of metabolite and lipid profiles in a segregating peach population associated with mealiness in *Prunus persica* (L.) Batsch. *Metabolites* 10:154 10.3390/metabo10040154 PMC7240955 32316167

[B40] Link H. (2000). Significance of flower and fruit thinning on fruit quality. *Plant Growth Regul.* 31 17–26.

[B41] Liu Y. Roof S. Ye Z. Barry C. van Tuinen A. Vrebalov J. (2004). Manipulation of light signal transduction as a means of modifying fruit nutritional quality in tomato. *Proc. Natl. Acad. Sci. U.S.A.* 101 9897–9902. 10.1073/pnas.0400935101 15178762 PMC470770

[B42] Lo Bianco R. Rieger M. (2002). Partitioning of sorbitol and sucrose catabolism within peach fruit. *J. Am. Soc. Hortic. Sci.* 127 115–121. 10.21273/JASHS.127.1.115

[B43] Lo Bianco R. Rieger M. Sung S. S. (1999). Activities of sucrose and sorbitol metabolizing enzymes in vegetative sinks of peach and correlation with sink growth rate. *J. Am. Soc. Hortic. Sci.* 124 381–388. 10.21273/jashs.124.4.381

[B44] Loewus F. A. Murthy P. P. N. (2000). myo-Inositol metabolism in plants. *Plant Sci.* 150 1–19. 10.1016/S0168-9452(99)00150-8

[B45] Lombardo V. A. Osorio S. Borsani J. Lauxmann M. A. Bustamante C. A. Budde C. O. (2011). Metabolic profiling during peach fruit development and ripening reveals the metabolic networks that underpin each developmental stage. *Plant Physiol.* 157 1696–1710. 10.1104/pp.111.186064 22021422 PMC3327199

[B46] Masia A. Zanchin A. Rascio N. Ramina A. (1992). Some biochemical and ultrastructural aspects of peach fruit development. *J. Am. Soc. Hortic. Sci.* 117 808–815. 10.21273/jashs.117.5.808

[B47] Mizutani F. Hirota R. Amano S. Hino A. Kadoya K. (1991). Changes in cyanogenic glycoside content and β-cyanoalanine synthase activity in flesh and seeds of Japanese plum (*Prunus salicina* Lindl.) during development. *J. Jpn. Soc. Hortic. Sci.* 59 863–867. 10.2503/jjshs.59.863

[B48] Moing A. Carbonne F. Rashad M. H. Gaudillère J. P. (1992). Carbon fluxes in mature peach leaves. *Plant Physiol.* 100 1878–1884. 10.1104/pp.100.4.1878 16653212 PMC1075879

[B49] Moing A. Svanella L. Rolin D. Gaudillère M. Gaudillère J. P. Monet R. (1998). Compositional changes during the fruit development of two peach cultivars differing in juice acidity. *J. Am. Soc. Hortic. Sci.* 123 770–775. 10.21273/jashs.123.5.770

[B50] Moriguchi T. Sanada T. Yamaki S. (1990). Seasonal fluctuation of some enzymes relating to sucrose and sorbitol metabolism in peach fruit. *J. Am. Soc. Hortic. Sci.* 115 278–281. 10.21273/JASHS.115.2.278

[B51] Myers S. C. Savelle A. T. Tustin D. S. Byers R. E. (2002). Partial flower thinning increases shoot growth, fruit size, and subsequent flower formation of peach. *HortScience* 37 647–650. 10.21273/hortsci.37.4.647

[B52] Nuñez C. Dupré G. Mujica K. Melet L. Meisel L. Almeida A. M. (2019). Thinning alters the expression of the PpeSUT1 and PpeSUT4 sugar transporter genes and the accumulation of translocated sugars in the fruits of an early season peach variety. *Plant Growth Regul.* 88 283–296. 10.1007/s10725-019-00507-0

[B53] Ognjanov V. Vujanić-Varga D. Mišić P. D. Verešbaranji I. Macet K. Tešović Ž, et al. (1995). Anatomical and biochemical studies of fruit development in peach. *Sci. Hortic.* 64 33–48. 10.1016/0304-4238(95)00825-9

[B54] Olmstead J. W. Iezzoni A. F. (2007). Genotypic differences in sweet cherry fruit size are primarily a function of cell number. *J. Am. Soc. Hort. Sci.* 132 697–703. 10.21273/JASHS.132.5.697

[B55] Pan L. Zeng W. Niu L. Lu Z. Liu H. Cui G. (2015). PpYUC11, a strong candidate gene for the stony hard phenotype in peach (*Prunus persica* L. Batsch), participates in IAA biosynthesis during fruit ripening. *J. Exp. Bot.* 66 7031–7044. 10.1093/jxb/erv400 26307136 PMC4765781

[B56] Pavel E. W. DeJong T. M. (1993). Source- and sink-limited growth periods of developing peach fruits indicated by relative growth rate analysis. *J. Am. Soc. Hortic. Sci.* 118 820–824. 10.21273/JASHS.118.6.820

[B57] Reighard G. L. Maffit W. Feng T. Souza F. B. M. Saski C. Pio R. (2018). Early thinning of peach fruit influences expression of cell growth and expansion genes. *Acta Hortic.* 1229 93–99. 10.17660/ActaHortic.2018.1229.15

[B58] Roch L. Prigent S. Klose H. Cakpo C.-B. Beauvoit B. Deborde C. (2020). Biomass composition explains fruit relative growth rate and discriminates climacteric from non-climacteric species. *J. Exp. Bot.* 71 5823–5836. 10.1093/jxb/eraa302 32592486 PMC7540837

[B59] Schluepmann H. Pellny T. van Dijken A. Smeekens S. Paul M. (2003). Trehalose 6-phosphate is indispensable for carbohydrate utilization and growth in *Arabidopsis thaliana*. *Proc. Natl. Acad. Sci. U.S.A.* 100 6849–6854. 10.1073/pnas.1132018100 12748379 PMC164535

[B60] Schmitzer V. Slatnar A. Mikulic-Petkovsek M. Veberic R. Krska B. Stampar F. (2011). Comparative study of primary and secondary metabolites in apricot (*Prunus armeniaca* L.) cultivars. *J. Sci. Food Agr.* 91 860–866. 10.1002/jsfa.4257 21384353

[B61] Scorza R. May L. G. Purnell B. Upchurch B. (1991). Differences in number and area of mesocarp cells between small- and large-fruited peach cultivars. *J. Am. Soc. Hortic. Sci.* 116 861–864. 10.21273/jashs.116.5.861

[B62] Seymour G. B. Østergaard L. Chapman N. H. Knapp S. Martin C. (2013). Fruit development and ripening. *Annu. Rev. Plant Biol.* 64 219–241. 10.1146/annurev-arplant-050312-120057 23394500

[B63] Singh R. Rastogi S. Dwivedi U. N. (2010). Phenylpropanoid metabolism in ripening fruits. *Compr. Rev. Food Sci. Food Saf.* 9 398–416. 10.1111/j.1541-4337.2010.00116.x 33467837

[B64] Sutton M. Doyle J. Chavez D. Malladi A. (2020). Optimizing fruit-thinning strategies in peach (*Prunus persica*) production. *Horticulturae* 6:41 10.3390/horticulturae6030041

[B65] Tonutti P. Casson P. Ramina A. (1991). Ethylene biosynthesis during peach fruit development. *J. Am. Soc. Hortic. Sci.* 116 274–279. 10.21273/JASHS.116.2.274

[B66] Vizzotto G. Pinton R. Varanini Z. Costa G. (1996). Sucrose accumulation in developing peach fruit. *Physiol. Plant.* 96 225–230. 10.1111/j.1399-3054.1996.tb00206.x

[B67] Volpe G. Lo Bianco R. Rieger M. (2008). Carbon autonomy of peach shoots determined by ^13^C-photoassimilate transport. *Tree Physiol.* 28 1805–1812. 10.1093/treephys/28.12.1805 19193563

[B68] Wu B. H. Quilot B. Génard M. Kervella J. Li S. H. (2005). Changes in sugar and organic acid concentrations during fruit maturation in peaches, *P. davidiana* and hybrids as analyzed by principal component analysis. *Sci. Hortic.* 103 429–439. 10.1016/j.scienta.2004.08.003

[B69] Wu T. Tao S. T. Zhang H. P. Song Y. Yao G. F. Zhang S. L. (2011). Effects of fruit thinning on fruit sugar accumulation and leaf photosynthetic characteristics of pear. *Acta Hortic. Sinica.* 38 2041–2048.

[B70] Zanchin A. Bonghi C. Casadoro G. Ramina A. (1994). Separation and cell enlargement development researches carried to the early division is restricted cycle. Four weeks after full bloom the final cell and endocarp appears number in the mesocarp to be fixed (Masia et al., 1992), and the subsequent by gro. *Int. J. Plant Sci.* 155 49–56.

[B71] Zhang C. Shen Z. Zhang Y. Han J. Ma R. Korir N. K. (2013). Cloning and expression of genes related to the sucrose-metabolizing enzymes and carbohydrate changes in peach. *Acta Physiol. Plant* 35 589–602. 10.1007/s11738-012-1100-1

[B72] Zheng B. Zhao L. Jiang X. Cherono S. Liu J. J. Ogutu C. (2021). Assessment of organic acid accumulation and its related genes in peach. *Food Chem.* 334 127567. 10.1016/j.foodchem.2020.127567 32707362

