# Supplementary figures and images for: Metabolite Fruit Profile Is Altered in Response to Source–Sink Imbalance and Can Be Used as an Early Predictor of Fruit Quality in Nectarine
Source: Front Plant Sci. 2021 Jan 8;11:604133. doi: 10.3389/fpls.2020.604133 (PMC7820367; doi:10.3389/fpls.2020.604133)

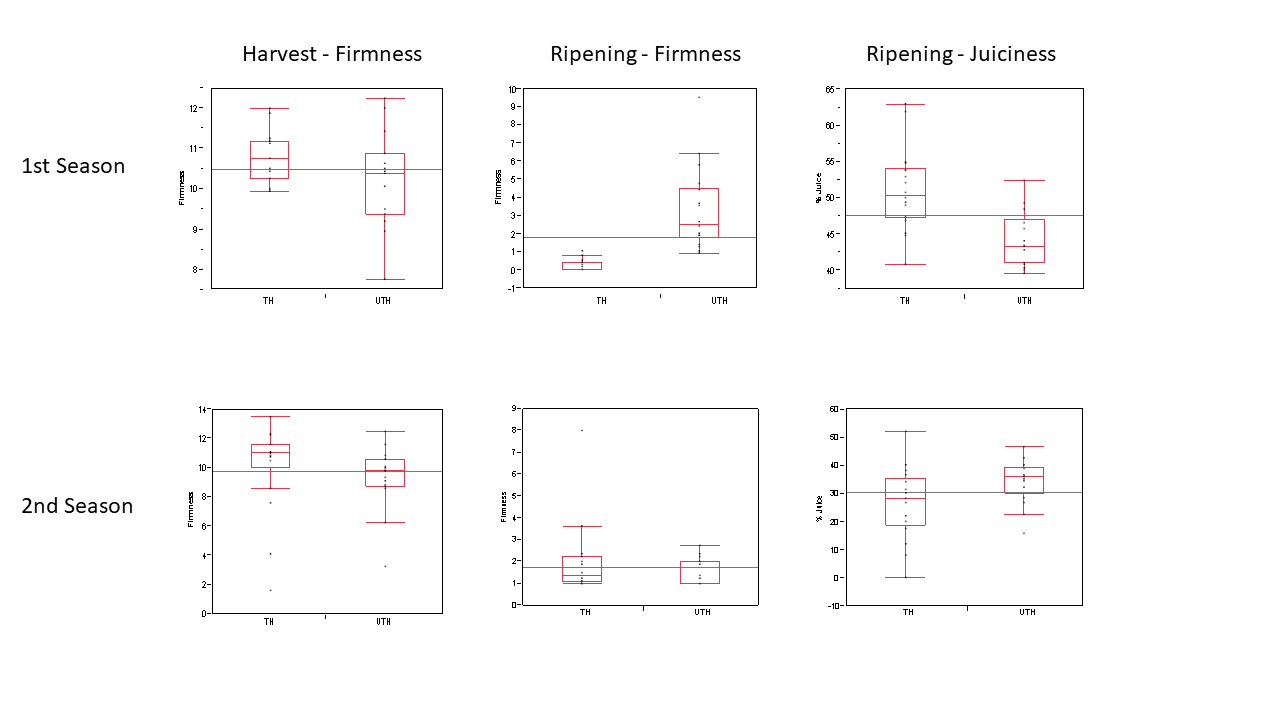

Supplement: Supplementary Figure 1 — Phenotypic analysis of “Magique” nectarines harvested from unthined (UTH) and thinned (TH) trees in both seasons. A boxplot analysis was performed to select the most similar fruits. In harvest, firmness was evaluated and in ripening firmness and juiciness were analyzed. [file Image_1.TIF]

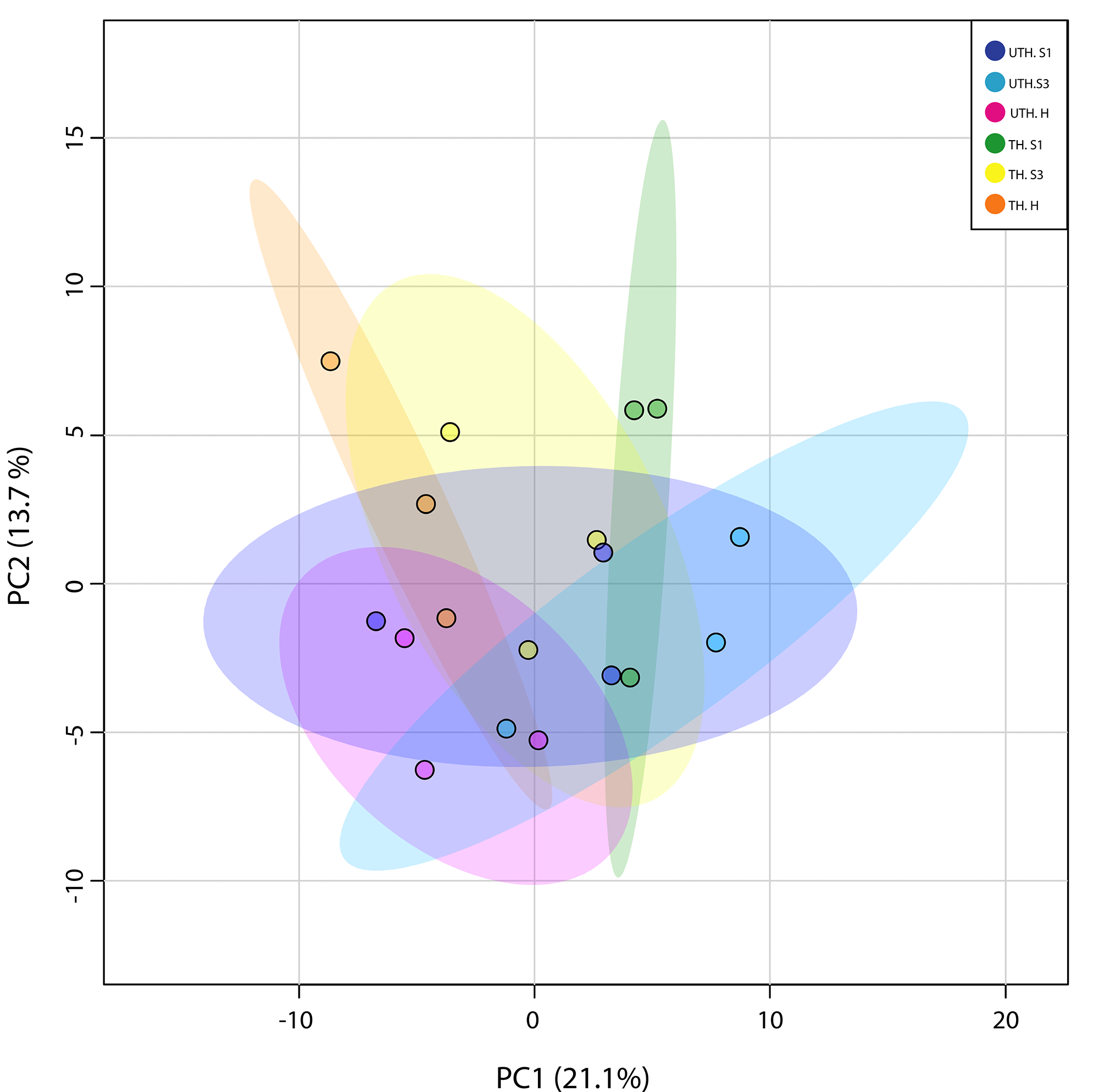

Supplement: Supplementary Figure 2 — Principal component analysis (PCA) of the 124 metabolites detected by GC-MS at S1, S3 and harvest (H) stage from unthinned (UTH) and thinned (TH) trees during the second season. The detected metabolites were employed as predictor variables, and the stages of development as a response variable. The panel shows the score plot where the variance explained of each component corresponded to 21.1% for PC1 and 13.7% for PC2. Blue circles represent S1 fruit samples from unthinned trees and green circles from thinned trees. Light blue circles represent S3 fruit samples from unthinned trees and yellow circles from unthinned trees. Pink circles represent harvested fruit samples from unthinned trees and orange circles from thinned trees. [file Image_2.TIF]

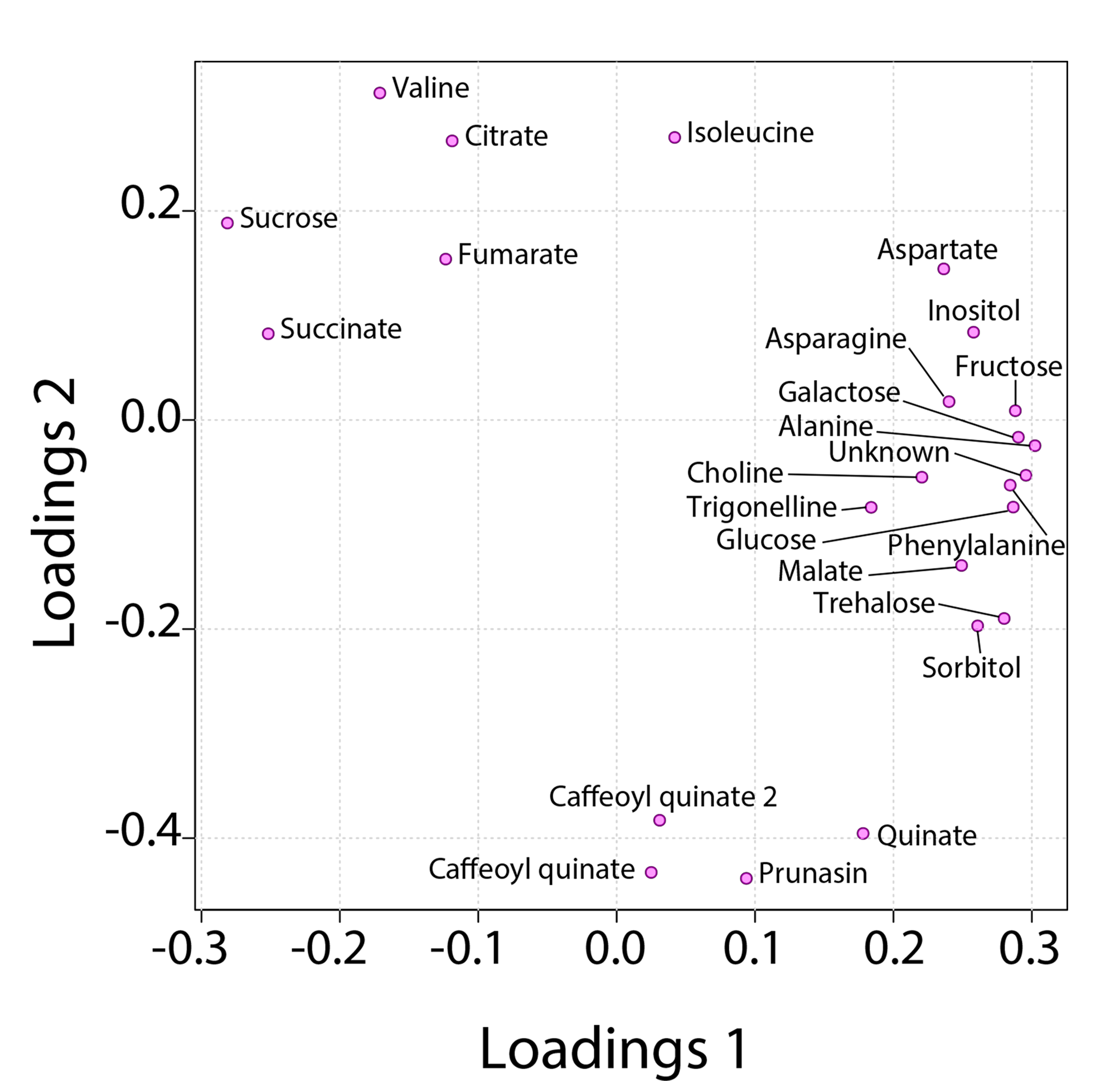

Supplement: Supplementary Figure 3 — Loadings plot between the selected PCs of Partial Least Square Analysis Discriminant Analysis (PLS-DA) of “Magique” nectarine metabolites detected using 1H-NMR for first season. [file Image_3.TIF]

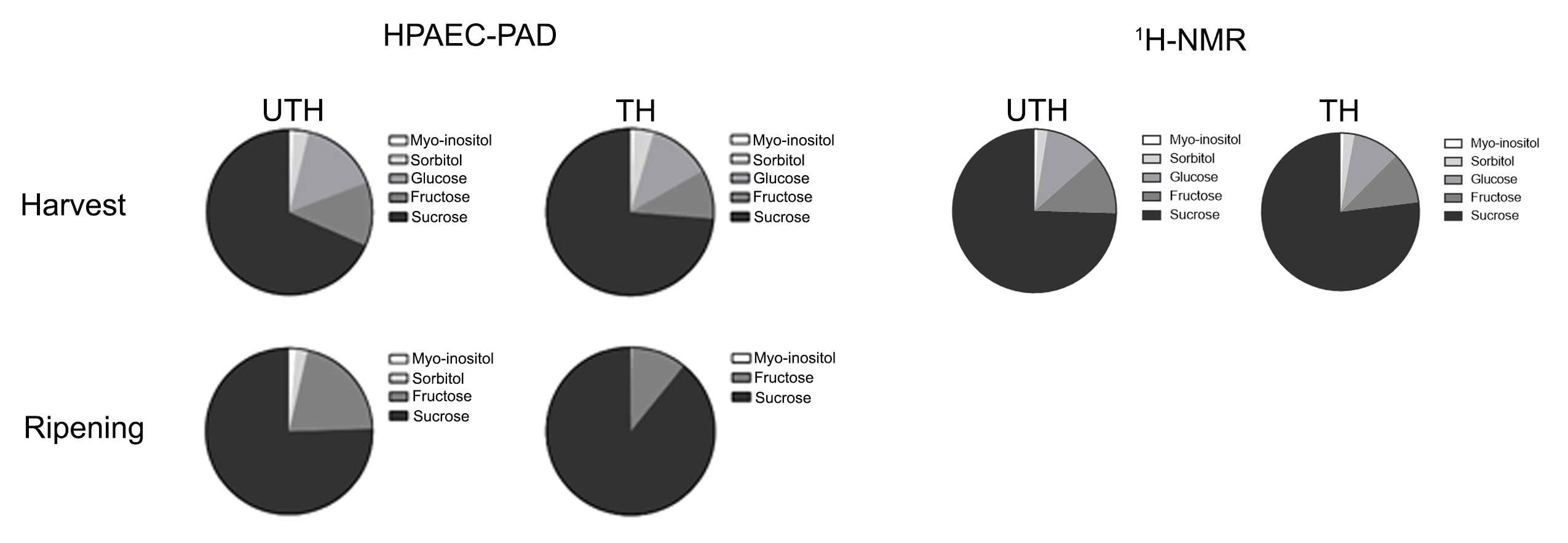

Supplement: Supplementary Figure 4 — Sugar proportions in the mesocarp of “Magique” nectarine fruit during the first season measured by HPAEC-PAD (harvest and ripening stages) and 1H-NMR (harvest stage). The total concentration of myo-inositol, sorbitol, glucose, fructose and sucrose together was considered as 100% and the proportion of each sugar was plotted. [file Image_4.TIF]
