# Supplementary material for: Metabolite Fruit Profile Is Altered in Response to Source–Sink Imbalance and Can Be Used as an Early Predictor of Fruit Quality in Nectarine
Source: Front Plant Sci. 2021 Jan 8;11:604133. doi: 10.3389/fpls.2020.604133 (PMC7820367; doi:10.3389/fpls.2020.604133)
Supplement: Supplementary Table 2 — Primers used for RT PCR analysis. [file Table_3.DOCX]

**Table S2:** Primers used for RTPCR analysis

| Primer name | Gene | Locus | Sequence |
| --- | --- | --- | --- |
| S1 | Dehydratation-responsive protein RD22 (RD22-like protein) | Prupe.1G475100 | F: 5’ AAAGGCGATGATGGGGAGAAGGT 3’  R: 5’ AAATGAAATGGCAGATGGGAATGG 3’ |
| S4 | Auxin-responsive protein IAA (Aux/IAA) | Prupe.8G232200 | F: 5’ CCAAGACCAACAAGGACAACA 3’  R: 5’ CATGCTCACCTTCACCA 3’ |
| TEF II | Translational elongation factor 2 | Prupe.4G138700 | F: 5’ GGTGTGACGATGAAGAGTGATG 3’  R: 5’ TGAAGGAGAGGGAAGGTGAAAG 3’ |
